# Identification of Thyroid Hormone Receptor Binding Sites and Target Genes Using ChIP-on-Chip in Developing Mouse Cerebellum

**DOI:** 10.1371/journal.pone.0004610

**Published:** 2009-02-25

**Authors:** Hongyan Dong, Carole L. Yauk, Andrea Rowan-Carroll, Seo-Hee You, R. Thomas Zoeller, Iain Lambert, Michael G. Wade

**Affiliations:** 1 Hazard Identification Division, EHSRB, Health Canada, Ottawa, Ontario, Canada; 2 Mechanistic Study Division, EHSRB, Health Canada, Ottawa, Ontario, Canada; 3 Molecular & Cellular Biology Program, University of Massachusetts, Amherst, Massachusetts, United States of America; 4 Department of Biology, Carleton University, Ottawa, Ontario, Canada; Katholieke Universiteit Leuven, Belgium

## Abstract

Thyroid hormone (TH) is critical to normal brain development, but the mechanisms operating in this process are poorly understood. We used chromatin immunoprecipitation to enrich regions of DNA bound to thyroid receptor beta (TRβ) of mouse cerebellum sampled on post natal day 15. Enriched target was hybridized to promoter microarrays (ChIP-on-chip) spanning −8 kb to +2 kb of the transcription start site (TSS) of 5000 genes. We identified 91 genes with TR binding sites. Roughly half of the sites were located in introns, while 30% were located within 1 kb upstream (5′) of the TSS. Of these genes, 83 with known function included genes involved in apoptosis, neurodevelopment, metabolism and signal transduction. Two genes, MBP and CD44, are known to contain TREs, providing validation of the system. This is the first report of TR binding for 81 of these genes. ChIP-on-chip results were confirmed for 10 of the 13 binding fragments using ChIP-PCR. The expression of 4 novel TH target genes was found to be correlated with TH levels in hyper/hypothyroid animals providing further support for TR binding. A TRβ binding site upstream of the coding region of myelin associated glycoprotein was demonstrated to be TH-responsive using a luciferase expression system. Motif searches did not identify any classic binding elements, indicating that not all TR binding sites conform to variations of the classic form. These findings provide mechanistic insight into impaired neurodevelopment resulting from TH deficiency and a rich bioinformatics resource for developing a better understanding of TR binding.

## Introduction

Thyroid hormone (TH) is essential for brain development in humans and animals [1]. The neonatal period of human development is particularly well-studied in part because of the disorder known as congenital hypothyroidism (CH) [2]. CH occurs at a rate of approximately 1 in 3,500 live births [3], though this may be increasing [4]. CH infants do not present early specific clinical features; therefore, only 10% of CH infants were diagnosed within the first month, 35% within 3 months, 70% within the first year, and 100% only after age 3 [5], [6] before neonatal screening for TH was implemented. The intellectual deficits resulting from this delayed diagnosis and treatment were profound. One meta-analysis found that the mean full-scale intelligence quotient (IQ) of 651 CH infants was 76 [7]. Moreover, the percentage of CH infants with an IQ above 85 was 78% when the diagnosis was made within 3 months of birth, 19% when it was made between 3 and 6 months, and 0% when diagnosed after 7 months of age [7], [8]. Thus, TH plays a major role in brain development and thyroid dysfunction is a major cause of mental retardation. This is also particularly important because a large number of environmental contaminants may impact thyroid function and/or thyroid hormone action [9]–[12].

The molecular mechanisms by which TH impacts brain development are becoming better understood. In general, it is postulated that many of the effects of TH are mediated by their receptors (TRs) – nuclear proteins that directly regulate gene expression [13]. Likewise, the neurodevelopmental events affected by TH are also becoming better understood. For example, TH appears to regulate fate specification of early cortical neurons [14], migration of cortical [15] and cerebellar [16] neurons, synaptogenesis[17], [18] and apoptosis [19], [20]. However, the specific genes that are directly regulated by TH through the TRs, and which account for TH effects on specific developmental events, are poorly characterized. Moreover, we know little about the DNA regulatory elements through which TRs exert their actions on gene regulation [21].

Identification of direct targets of TH in the developing brain has proven difficult, but perhaps for predictable reasons. Specifically, we [19], [22] and others [23], [24] have attempted to identify TH-responsive genes in the developing brain using a variety of “functional genomics” approaches. All of these reports employed an RNA-based approach. However, a significant theoretical weakness in this approach is that the cellular phenotype of the hypothyroid brain in development is significantly different from that of the euthyroid brain. For example, the hypothyroid brain has considerably more astrocytes and fewer oligodendrocytes in areas of white matter [25], [26]. Therefore, it is likely that a large number of expressed sequences that differ in abundance when thyroid hormone levels are manipulated during development reflect differences in cellular phenotype at the time of sacrifice.

Considering the importance of TH to brain development and the challenge of identifying direct gene targets of TH action, we reasoned that chromatin immunoprecipitation combined with DNA microarray analysis (ChIP-on-chip) would be an effective approach to identify direct gene targets of TH. Moreover, we chose to focus on the neonatal (postnatal day (PND) 15) cerebellum because it is a well studied target of TH action in development [27]. Circulating TH levels reach a peak at PND 15 [28], [29], the critical time point marked by the onset of active myelination and synapses refinement in Purkinje cells [25], [30], [31]. We employed custom DNA microarrays that contained probes covering 10 kb of genomic sequences flanking the transcription start site (TSS) of 5000 genes selected from our pervious microarray studies and related literatures. The use of microarrays containing selected genes tends to limit the potential for false positives that would invariably occur with arrays containing the entire genome and increases the number of probes per gene, thus improving sensitivity and resolution. This study represents the first large-scale approach to identify direct gene targets of TH action in the developing brain.

## Results

### Assessing specificity of polyclonal antibody and ChIP protocol

**Figure 1:**
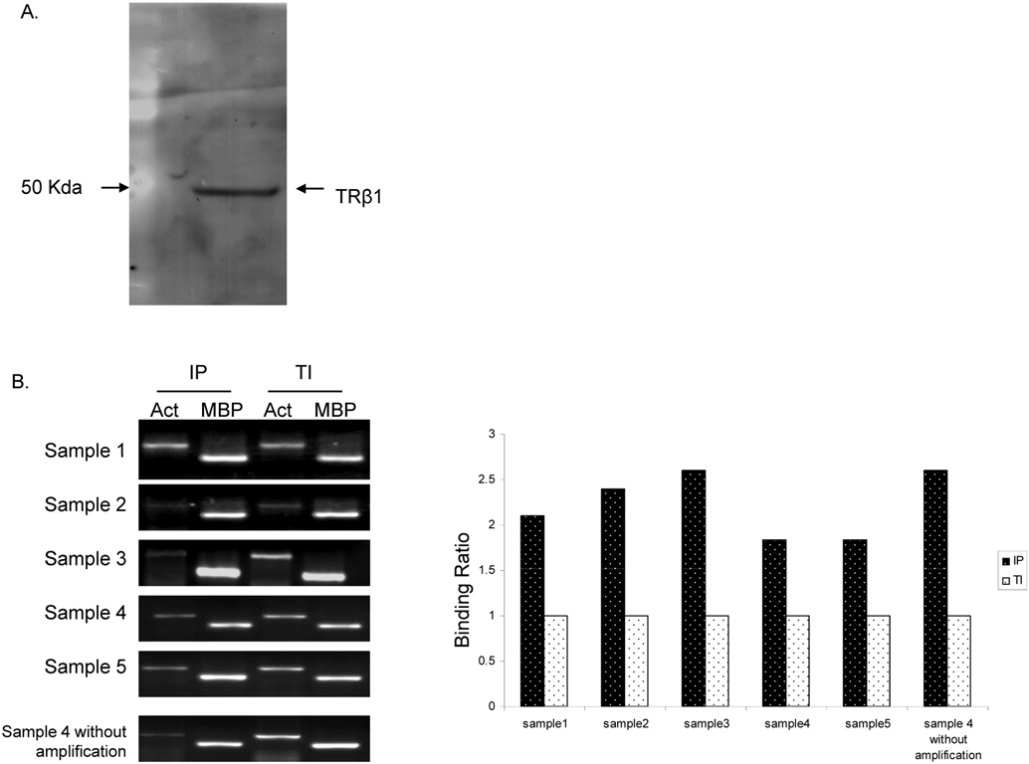
Assessing specificity of antibody and ChIP protocol. A. Characterization of TRβ antibody with the GH3 cell line. A single 52 kDa band was detected with Western blot. B. Characterization of the enrichment of MBP-TRE in all cerebellum samples. Enrichment of fragments from the promoter region of β-actin and MBP was examined with PCR using IP and TI DNA amplified with WGA kit. One representative sample in the bottom shows the enrichment in non-amplified DNA. The right panel shows the enrichment ratio of MBP to β-actin calculated using quantified band intensities.

The TRβ-1 antibody used for ChIP in the current study showed high specificity as indicated by Western blot analysis using GH3 cell extracts (Figure 1 A). A single band of 52 kDa was detected as expected. Immunoprecipitated (IP) DNA and total input (TI) DNA were amplified and evaluated by confirming the enrichment of the well-characterized TRE in the promoter of myelin basic protein (MBP) [32] by PCR before samples were hybridized to DNA microarrays. Figure 1 B shows clear enrichment of MBP-TRE (relative to β-actin) in amplified ChIP DNA of all 5 cerebella of male mice at PND 15.

### Identification of TRβ binding sites

**Figure 2:**
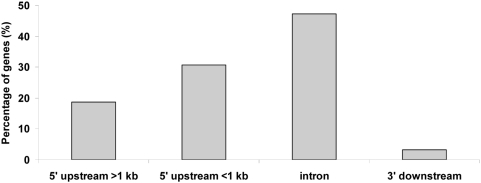
Distribution of genomic locations of binding sites of 91 genes. The mid point of each probe was used to calculate the distance to the closest gene.

**Figure 3:**
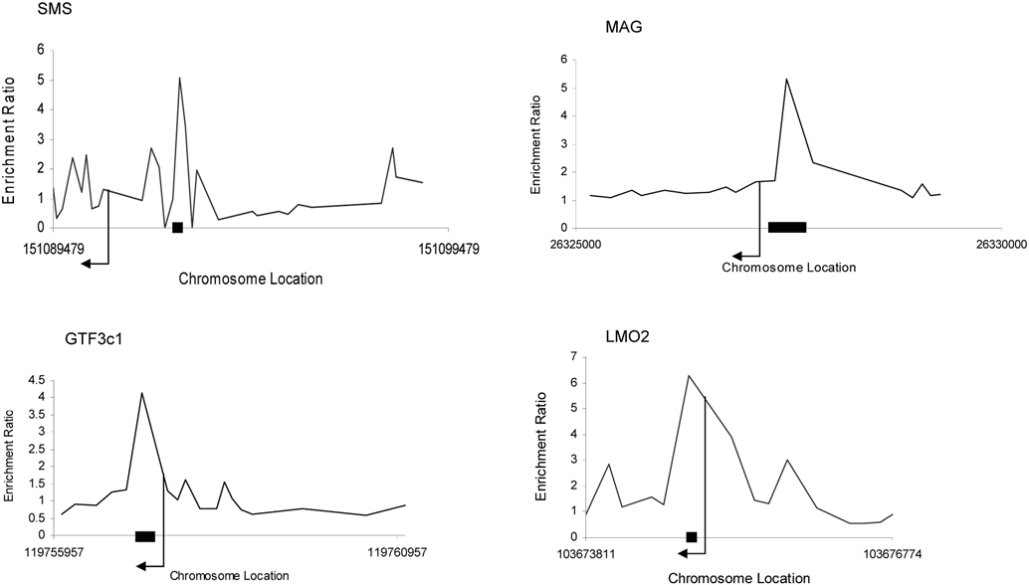
Examples of TRβ binding activities identified with ChIP-on-chip. The plots show enrichment ratios for all probes within a genomic region (IP versus TI DNA). Chromosomal positions are from NCBI build 34 of the mouse genome. The start and direction of transcription are noted by arrows. Black bars on the X axis indicate the fragments whose enrichment was confirmed with PCR.

Five cerebella of male mice, shown in Figure 1 B, were analysed using the Agilent custom promoter arrays that contained probes covering 10 kb of genomic sequences flanking the TSS of 5000 selected genes. Complete microarray data are available at MIAMExpress (http://www.ebi.ac.uk/miamexpress/cgi-bin/mx.cgi; accession number E-MEXP-1801). The genes identified with the Chip Analytics software as enriched in at least 3 samples are listed in Table 1, along with the genomic location of the binding sites, enrichment log ratio, and general biological function. As shown in Figure 2, approximately half of the binding sites were located in introns, while about 30% of them were located within 1 kb upstream (5′) of the TSS of their associated gene. Figure 3 shows examples of ChIP enrichment data for 4 representative genes. The plots show enrichment ratios along with chromosomal positions (NCBI build 34) in the mouse genome. Arrows indicate both the TSS and direction of transcription.

**Table 1:** Thyroid Hormone Direct Target Genes Identified by ChIP-on-chip.

Acc. No.	Gene Symbol	Probe Location	Enrichment Log ratio					Median
			Rep1	Rep2	Rep3	Rep4	Rep5	
*Apoptosis*								
NM_028133	Egln3	intron	2.7	1.59	1.77	2.28	1.63	1.77
NM_178931	Tnfrsf14	intron	1.92	1.8	2.45	2.01	1.36	1.92
NM_010589	Jak3	2.2 kb 5′	4.32	3.22	1.7	2.02	1.32	2.02
NM_008353	Il12rb1	6.7 kb 5′	2.88	1.93	2.17	2.1	1.76	2.1
NM_007466	Api5	intron	2.88	4.52	0.29	2.27	0.74	2.27
NM_175445	Rassf2	0.7 kb 5′	1.58	2.57	2.76	2.46	2.2	2.46
NM_013929	Siva	0.7 kb 5′	2.24	2.7	2.52	3.06	2.91	2.7
NM_008841	Pik3r2	intron	5.28	3.15	1.78	2.92	1.85	2.92
NM_009746	Bcl7c	intron	2.39	3.1	4.56	5.1	1.28	3.1
NM_022654	Lrdd	0.6 kb 3′	6.85	3.83	6.48	3.09	4.95	4.95
*Cell cycle*								
NM_133655	Cd81	intron	1.56	1.33	2.2	1.87	1.37	1.56
NM_023117	Cdc25b	0.1 kb 5′	1.44	3.03	1.73	1.7	1.96	1.73
NM_030241	Setd8	0.2 kb 5′	2.53	4.06	1.44	1.89	1.12	1.89
*Teeth or bone development*								
BC005460	Ncl	0.4 kb 5′	1.95	1.98	1.38	1.26	1.78	1.78
NM_010514	Igf2	intron	3.46	2.83	1.39	1.91	1.53	1.91
NM_008275	Hoxd13	1.2 kb 5′	2.26	2.73	2.3	6.33	2.42	2.42
NM_145925	Pttg1ip	intron	3.66	3.14	3.39	3.93	0.87	3.39
*RNA or DNA process*								
AK077829	Rad51ap1	intron	5.02	2.34	4.68	2.72	2.48	2.72
AK077107	Frg1	0.2 kb 5′	1.92	1.48	3.5	2.56	1.69	1.92
NM_182650	Hnrnpa2b1	0.5 kb 5′	3.13	4.89	5.08	1.66	2.03	3.13
*Metabolism*								
NM_007823	Cyp4b1	intron	1.35	2.02	1.51	2	1.67	1.67
NM_011977	Slc27a1	intron	2.45	2.19	0.99	1.67	1.54	1.67
NM_025578	Mrps25	0.2 kb 5′	3.64	1.74	2.86	1.37	1.75	1.75
NM_153803	Glb1l2	intron	7.42	1.29	4.57	1.93	0.86	1.93
NM_027868	Slc41a3	intron	2.08	2.19	2.54	2.07	1.66	2.08
NM_008131	Glul	intron	1.85	2.67	2.14	2.14	2.11	2.14
NM_175311	Zfp513	intron	2.14	1.95	1.85	3.13	2.57	2.14
NM_010361	Gstt2	3.4 kb 5′	1.73	4.32	1.71	2.44	2.31	2.31
NM_026796	Smyd2	intron	2.06	7.09	2.4	2.79	1.29	2.4
NM_011376	Sim1	1.5 kb 5′	1.9	1.51	5.08	3.44	2.45	2.45
NM_025802	Pnpla2	intron	5.57	3.97	2.47	2.28	1.06	2.47
NM_008972	Ptma	intron	2.89	3.74	2.47	2.26	2.02	2.47
NM_146188	Kctd15	0.8 kb 5′	3.13	3.78	2.54	2.18	1.77	2.54
NM_008673	Nat1	6.2 kb 5′	2.55	3.51	2.65	1.9	1.53	2.55
NM_020013	Fgf21	intron	2.09	2.56	5.73	2.78	2.37	2.56
NM_023122	Gpm6b	intron	5.29	4.33	2.8	1.1	2.77	2.8
NM_019652	ASNA1	intron	4.7	5.65	2.84	2.3	2.41	2.84
NM_011506	Sucla2	intron	4.45	2.93	5.47	2.52	1.56	2.93
NM_025286	Slc31a2	intron	3.29	2.87	1.93	4.71	3.66	3.29
NM_011969	Psma7	intron	2.84	3.45	3.3	3.27	3.38	3.3
NM_007945	Eps8	0.6 kb 5′	2.82	3.95	4.55	2.75	4.55	3.95
*Nerve development*								
NM_027180	Centd2	3.8 kb 5′	3.98	1.9	1.3	1.58	0.86	1.58
NM_010419	Hes5	7.8 kb 5′	1.57	1.68	2.17	1.77	1.82	1.77
NM_008500	Lhx6	0.1 kb 5′	5.12	5.26	1.83	1.82	1.26	1.83
NM_008630	Mt2	0.8 kb 5′	1.85	2.85	1.99	1.4	0.85	1.85
NM_009851	CD44	intron	1.91	2.1	1.73	2.08	1.92	1.92
AF113001	Ncor2	0.7 kb 5′	1.34	3.35	4.19	1.92	1.54	1.92
NM_207682	Kif1b	0.5 kb 5′	1.16	1.96	2.89	3.4	1.17	1.96
NM_010758	Mag	0.4 kb 5′	1.63	1.86	2	2.64	2.41	2
NM_001029873	Unc13a	4.5 kb 5′	4.32	3.22	1.7	2.02	1.32	2.02
NM_021716	Fign	0.8 kb 5′	1.81	2.08	2.32	3.55	1.6	2.08
NM_010777	Mbp	intron	2.21	1.92	2.17	2.13	1.67	2.13
NM_010710	Lhx2	0.6 kb 5′	3.29	2.52	2.17	2.1	1.28	2.17
NM_009800	Car11	0.2 kb 3′	1.27	2.18	5.11	3.25	1.5	2.18
NM_009849	Entpd2	intron	2.19	2.18	3.25	2.92	1.84	2.19
NM_001025245	Mbp (Goli)	intron	1.31	3.68	3.34	2.19	1.17	2.19
NM_009501	Vax1	1.4 kb 5′	3.57	2.3	1.79	2.91	1.62	2.3
NM_009718	Neurog2	6.6 kb 5′	3.16	2.42	2.32	1.87	1.58	2.32
NM_019446	BarhL1	0.9 kb 5′	2.71	2.53	1.97	2.35	2.06	2.35
NM_008505	Lmo2	intron	2.04	2.43	2.38	2.65	2.04	2.38
NM_175750	Plxna4	2.8 kb 5′	4.07	2.93	5.27	2.12	1.91	2.93
NM_013703	Vldlr	intron	2.28	4.16	4.53	3.24	2.3	3.24
NM_201618	Gnas	intron	3.51	3.63	3.78	4.64	2.18	3.63
NM_010700	Ldlr	intron	2.61	4.31	3.51	3.73	4.62	3.73
NM_009214	Sms	1.0 kb 5′	2.06	2.34	4.41	2.45	1.86	2.34
*Signal transduction*								
NM_010098	Opn3	intron	2.13	2.78	2.67	2.25	1.97	2.25
NM_008772	P2ry1	intron	1.32	3.29	3.83	2.46	1.11	2.46
NM_183408	Pde4a	intron	2.56	3.38	2.52	2.14	2.02	2.52
NM_011951	Mapk14	0.1 kb 5′	2.94	2.12	6.09	4.14	1.55	2.94
NM_028041	DDX54	0.1 kb 5′	3.2	3.92	1.94	3.4	2.02	3.2
NM_025569	Mgst3	0.2 kb 5′	3.38	5.04	3.28	2.46	2.35	3.28
NM_009633	Adra2b	0.6 kb 5′	1.86	3.51	6.38	3.72	0.9	3.51
NM_011305	Rxra	0.8 kb 5′	1.37	1.67	1.93	1.42	1.71	1.67
NM_207239	Gtf3c1	intron	2.05	2.08	2.56	2.06	2.18	2.08
NM_016974	Dbp	0.5 kb 5′	1.27	2.18	5.11	3.25	1.5	2.18
NM_178622	Suds3	4.7 kb 5′	1.81	2.43	6.34	3.15	1.3	2.43
NM_172913	Tox3/Tnrc9	2.8 kb 5′	2.66	3.95	1.72	3.63	1.94	2.66
NM_008781	PAX3	7.2 kb 5′	3.17	4.13	1.76	2.88	1.67	2.88
NM_007624	Cbx3	0.3 kb 5′	3.13	4.89	5.08	1.66	2.03	3.13
NM_010827	Msc	0.3 kb 5′	3.88	2.63	2.66	41.3	3.38	3.38
NM_008093	Gata5	intron	3.89	3.66	5.23	1.91	2.11	3.66
NM_026646	Slc25a22	0.3 kb 5′	6.85	3.83	6.48	3.09	4.95	4.95
*Sperm function*								
NM_018808	DNAjb1	intron	1.69	2.12	3.91	1.94	1.21	1.94
NM_153144	Ggnbp2	intron	1.28	2.1	3.33	2.22	1.69	2.1
*Unknown*								
NM_182930	Plekha6	7.4 kb 5′	2.35	2.53	1.77	1.29	1.32	1.77
NM_028596	Riken	intron	2.23	2.03	1.87	1.71	1.45	1.87
NM_198628	GM711	intron	1.28	1.88	5.24	1.88	0.99	1.88
BC085130	MKIAA4027	intron	4.26	2.01	2.16	1.57	0.63	2.01
NM_028696	Obfc2a	intron	4.03	2.44	2.3	2.37	1.8	2.37
NM_026626	Efcab2	intron	2.31	3.2	3.98	2.49	1.88	2.49
NM_001101503	Riken	1.5 kb 3′	2.93	3.14	2.69	3.93	1.59	2.93
NM_144558	BIVM	0.8 kb 5′	1.65	2.03	3.07	3.12	3.74	3.07

### Validation of the binding sites with ChIP-PCR

**Figure 4:**
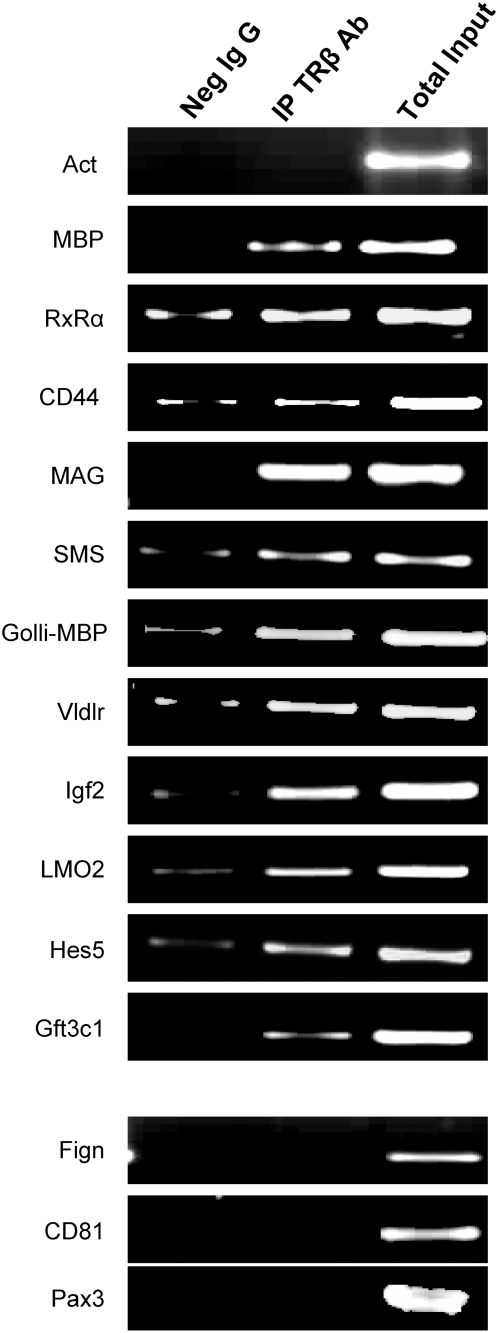
Confirmation of enriched genes identified using ChIP-on-chip with 13 randomly selected genes by PCR in independently prepared amplified IgG-IP, TRβ-IP and TI DNAs pool of 2 samples.

In order to verify the robustness of binding sites found with ChIP-on-chip, we randomly selected and analyzed 13 binding sites for confirmation of their enrichment using PCR. β-actin was used as a negative control, and MBP-TRE was used as a positive control. Amplified TRβ-IP or IgG-IP DNAs along with TI-DNAs from 2 cerebella were pooled, with equal quantities of DNA from each sample, and used as template for PCR. Ten of the 13 genes were confirmed to be enriched with TRβ binding as shown in Figure 4. Of the 10 confirmed genes, CD44 and VLDLR have previously been reported as TH responsive genes [29], [30]. In addition to the well-known TRE sequence in the promoter region of MBP, we also identified a second enrichment site in the first intron of Golli-MBP [31]. As 3 (Fign, CD81and Pax3) of 13 binding sites tested were not confirmed by PCR, these data suggest the analysis has false discovery rate of approximately 23% (3/13).

### Expression of novel TH target genes in TH modulated animal models

**Figure 5:**
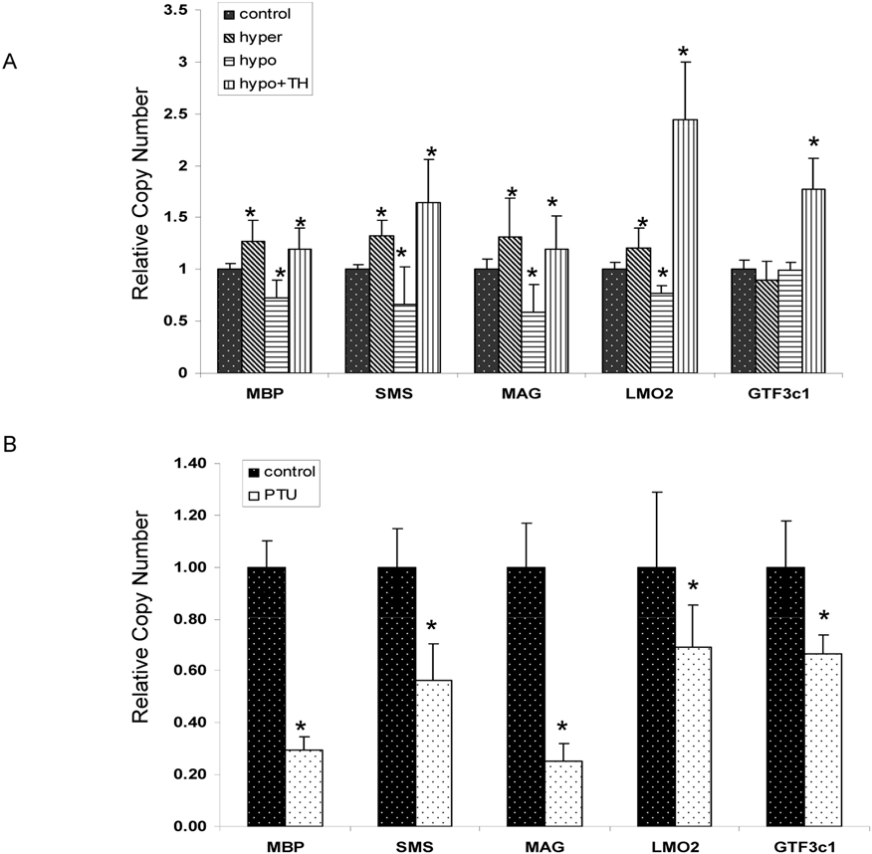
The expression of novel thyroid responsive genes in hypothyroid or hyperthyroid mouse models. A. MMI/perchlorate induced hypothyroid, hyperthyroid or hypothyroid/replacement animal models. B. PTU induced hypothyroid animal models. RT-PCR was performed with RNA extracted from cerebellum on PND15 (n = 5). * Significantly different from control (*p*<0.05).

Among the 10 ChIP-PCR validated genes, SMS (Spermine Synthase), LMO2 (Lim domain only 2) and GTF3c1 (General Transcript Factor 3c1) were selected for further assessment of TH regulation using *in vivo* models; this selection was based on the paucity of literature regarding TH control of their expression. In addition, myelin associated glycoprotein (MAG) was also selected for further analysis as it has been shown to be altered by TH status but no TRE has been described for this gene [32]. The expression of MBP was, again, used as a positive control for TH-induced gene regulation in the various animal models. Serum T4 levels in 6-propyl thiouracil (PTU) -or mercapto-methylimidazole (MMI)/perchlorate-treated pups were significantly reduced while TH injected pups were shown to have significantly elevated serum TH levels compared with control pups. The serum T4 levels in TH replacement pups rendered hypothyroid by MMI/perchlorate were approximately equivalent to control levels (Table S1). The expression of MBP varied with TH level (Figure 5 A and B). Similar to MBP, the expression of SMS, MAG and LMO2 were significantly changed corresponding with the TH levels. A significant change in expression of GTF3c1 was not found in TH injected pups or those rendered hypothyroid with MMI/perchlorate treatment, but expression was elevated almost 2 fold (*p*<0.05) in cerebella of hypothyroid/replacement group and reduced to almost 0.75 fold (*p*<0.05) in PTU-treated pups. The discrepancy in gene expression in these two models may be explained by the different mechanism through which these compounds act to induce hypothyroidism. PTU inhibits TH synthesis by inhibition of thyroperoxidase (TPO) and decreasing the activity of deiodinases, while MMI/perchlorate acts by blocking iodine transportation and enhancing hepatic catabolism [33].

### Characterization of TRβ binding region in promoter of MAG

**Figure 6:**
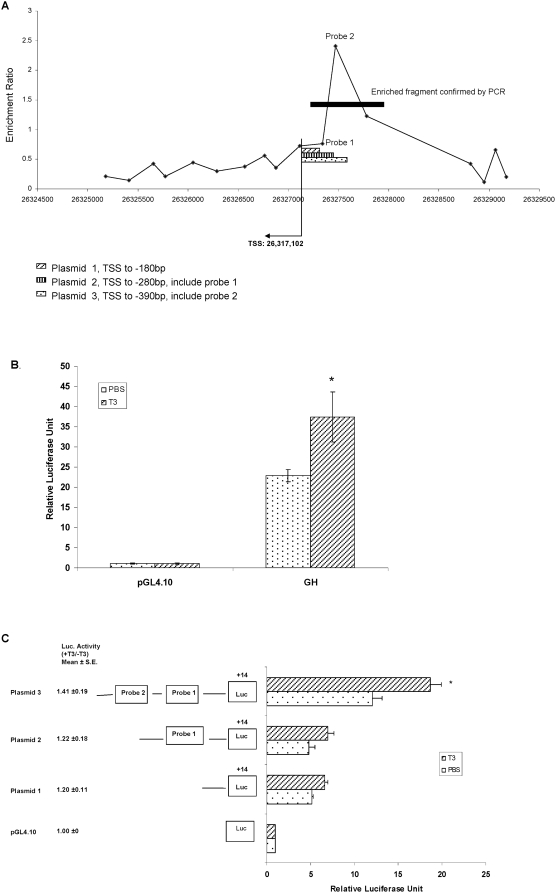
The transcriptional activity of the MAG promoter examined with the luciferase reporter assay. A. The location of the 3 truncated fragments used to build the reporter constructs. The PCR-confirmed ChIP enriched fragment is indicated by the black bar. The TSS and direction is indicated with an arrow. B and C. Transcriptional activity of GH reporter construct or MAG promoter reporter constructs induced by TH. The reporter constructs and empty vector were co-transfected into GH3 cells with pRL-TK (as a transfection efficiency control). T3 (10^−8^ M) was added after 24 hrs. Firefly luciferase expression was normalized to renilla luciferase from the pRL-TK plasmid. Values are mean±S.E. (n = 3). *indicates *p*<0.05.

As described above, the TRβ binding site in the promoter region of MAG was confirmed with both ChIP-on-Chip and ChIP-PCR. There were no obvious candidate TREs, based on the degenerate consensus sequence, across this binding region. To further characterize the TRE sequence in this region, we constructed a series of truncated MAG-promoter reporter plasmids (shown in Figure 6 A). The reporter system was validated by examining the effect of T3 treatment on transcriptional activity of the Growth Hormone (GH) TRE sequence cloned into the reporter vector. T3 induced GH transcriptional activity by 1.6-fold (Figure 6 B). T3 also increased transcriptional activity of a MAG-promoter reporter plasmid 3 containing a fragment from −390 bp to the TSS, by 1.4-fold. Deletion of the 110 bp fragment from −390 to −280 bp resulted in a decrease in the basal transcription level; transcription activity was induced by only 1.2 fold with T3 treatment (Figure 6 C). These results indicate that the binding site spanning a region of 110 bp (−390 to −280) may include the potential TREs.

## Discussion

This is the first report of a relatively large-scale approach to identify direct TH gene targets in the developing mouse brain. Using ChIP-on-chip approach, we identified TRβ binding fragments corresponding to 91 genes in the cerebellum of male mice at PND 15. Approximately half of these sites are located within introns, and 30% are located within 1 kb upstream of the TSS. Several of these genes are known direct targets of TH action (CD44, MBP and VLDLR), indicating that our strategy was capable of identifying known targets. In addition to confirming the classical TRE in the MBP promoter [34], we identified a novel TR binding site well upstream of the classic MBP TSS, located within the first intron of an alternate transcript of this gene known as Golli MBP [35]. Golli-MBP is expressed not only in myelin-forming cells, but also in neurons in the CNS and peripheral nervous system, and in macrophage and T-cells of the immune system [36], [37]. Direct TR regulation of Golli-MBP indicates the important role of TH in myelination and migration of oligodendrocytes, in addition to its role in the immune system. These findings provide a first glimpse at the specific gene targets through which TH controls cerebellar histogenesis in the mouse.

We found that a large proportion (47%) of the TR binding sites are located within introns, suggesting that transcriptional control by TR could be exerted at sites downstream of the TSS. It has long been recognized that active TREs can reside within introns of TH responsive genes. The TH-regulated expression of GH is mediated through both a TRE in the regulatory region upstream of the TSS [38] and a site with higher affinity for TR within the third intron of the gene [39]. Similarly, the expression of hepatic carnitine palmitoyltransferase-I alpha, an enzyme involved in fatty acid metabolism, is regulated by TR action at a TRE located in the first intron [40]. Intronic locations of transcriptional regulatory sites appear to be common for a variety of nuclear receptors including the androgen receptor [41], glucocorticoid receptor [42], vitamin D3 [43] and PPAR gamma [44]. Likewise, between 38 and 47% of estrogen receptor binding sites are located within introns [45], [46].

TR binding sites identified with ChIP-on-chip were analyzed independently with ChIP-PCR for 13 randomly selected genes. Ten of these sites were confirmed as enriched by TRβ ChIP, suggesting a false positive rate of approximately 23% for our approach. ChIP-on-chip is a novel method to study chromatin – DNA interactions over the entire genome. Our results are not unusual given that previous data from ChIP-on-chip studies have reported false positive rates as high as 30% [47]. There are a number of confounding variables, including purity of ChIP DNA, amplification methods and resolution of probes for each gene. It is also probable that the list of enriched genes identified by ChIP-on-chip is highly dependent on the algorithm applied (normalization, statistics to identify enriched regions, criteria for calling a site significant, etc.) which may influence the rate of false positives and false negatives.

Identified as one of novel targets of TH regulation, SMS is essential for transforming spermidine to spermine, which are ubiquitous cellular components that play critical roles in cellular physiology [48]. Polyamines are required for numerous cellular processes including transcription, translation and modulation of ion channel activities[49], [50] Deficiency of SMS in mice is associated with deafness, inner ear abnormalities, and hyperactivity, while congenital deficiency in humans is associated with mental retardation and cerebellar abnormalities [51]. All of these phenotypes are common manifestations of developmental hypothyroidism [52]. In the current study we identified TR binding sites in the promoter region of SMS. Moreover, TH positively regulated the expression of SMS in both mouse models of TH disruption. Microarray analyses of cerebellum transcript levels in mice or rats treated with a low concentration of PTU showed that SMS expression was reduced in hypothyroid mice of both sexes, but only in male hypothyroid rats (in preparation). Therefore, down-regulation of SMS, causing decreased production of polyamines, may be one mechanism that leads to neurodevelopmental aberrations in the offspring of hypothyroid dams. More work is required to investigate this pathway and its response to TH in the brain.

Among the other novel TH target genes selected for further analysis, LMO2 is a member of a family of genes encoding Lim-only proteins, which are speculated to affect the development of the mouse CNS because of their high expression in this region [53]–[55]. Using both ChIP-on-chip and ChIP-PCR, we found that TR bound to a LMO2 intron, and our results suggest that LMO2 expression is regulated by TH in our animal models. GTF3c1 is a transcription factor required for the regulation of genes transcribed by RNA polymerase III [56]. Our results indicate a TR binding site in the first intron of this gene and that the expression of GTF3c1 is upregulated in the cerebellum of hypothyroid animals with TH replacement (Figure 5). However, the reduced expression was found in hypothyroid animals induced by PTU, but not in animals induced by MMI/perchlorate. This suggests that factors other than TH may prevent the reduction in GTF3c1 expression. A recent study examining hepatic genome-wide expression in wild type and TR knock-out mice treated with TH or goitrogen found that, like GTF3c1, roughly half of all genes that showed positive regulation by TH excess, exhibited no response to TH deficiency [57]. Although that study did not attempt to determine the presence of genomic TR binding sites that could potentially regulate the expression of these genes, the results suggest that the pattern of response of GTF3c1 expression in the cerebella of TH-manipulated animals may not be uncommon for TH regulated genes.

MAG is a quantitatively minor component of isolated myelin that functions in glia-axon interactions [58]. The abnormal expression of MAG in hypo/hyperthyroidism has previously been reported in several studies [59]–[61], but there is little information on how its expression is directly affected by TH. The current study shows that TRβ binds in the region between −280 to −390 bp, which significantly influences the transcription activity. A search using an internet based tool to identify TREs based on classical descriptions (TESS: Transcription Element Search System [62] : located at http://www.cbil.upenn.edu/cgi-bin/tess/tess; ) failed to identify any of the classic TRE forms (DR4, DR6, palindromic form or inverted palindrome), suggesting that the sequence of the binding site is novel. We found a half TRE (AGGTCA) in this region, but alteration of this sequence to AGATCA by site-directed mutagenesis had no effect on the transcriptional activity *in vitro* (data not shown). This suggests that TRβ may not bind to the half TRE and that this specific sequence has no functional significance. The expression of MAG in our animal models is correlated with TH level suggesting that the presence of a TR binding site in the regulatory region of the gene is genuine. Further analyses will be needed to characterize the nature and sequence of the TR regulatory element in this gene, but current findings indicate that not all TREs will conform to variations of the classic forms.

In conclusion, we have identified TR binding sites associated with 91 genes in the developing mouse cerebellum. Binding sites may be located in different genomic contexts (both upstream and downstream of genes, and in introns) and may not adhere to conventional sequence models. As TR binding suggests the presence of consensus thyroid regulatory elements and, hence, direct TH regulation of these genes, the current study provides support for the role of products of these genes in thyroid hormone-directed neurodevelopment.

## Materials and Methods

### Animals and tissues collection

#### ChIP-on-chip

All animal handling procedures adhered to the Canadian Council on Animal Care guidelines and were approved by the Health Canada Animal Care Committee prior to the initiation of the study. Nulliparous, sexually mature C57BL/6 mice were purchased from Charles River (St. Constant, QC, Canada) and were housed individually (males) or in pairs (females) in hanging polycarbonate cages under a 12∶12 hrs light-dark cycle at 23°C with food (Purina rodent chow 5010; Ralston-Purina, MO) and water available *ad libitum*. After 2 weeks acclimation to the facility, breeding was initiated by transferring 2 females to each male cage. Females were examined within 1 hr of light cycle for the presence of a vaginal plug as an indication of pregnancy. Plug positive females were weighed and transferred to separate cages, where they were housed singly and weighed 10 days post coitus to confirm pregnancy. Dams were allowed to litter naturally (day of birth = PND 1) and numbers of pups per litter were not adjusted. On PND 15, pups were sacrificed by decapitation and cerebellum was removed, immediately frozen in liquid nitrogen and stored at −80°C.

#### PTU hypothyroid model

Pregnant C57BL/6 mice were supplied *ad libitum* with water containing diet cherry Kool-aid (Kraft Inc., ON, Canada) with or without 0.1% PTU (Sigma-Aldrich, Oakville, ON, Canada) from gestation day 13 to PND 15. On PND 15, 5 male pups from different litters were sacrificed by decapitation and cerebellum was collected and stored at −80°C. At least one littermate of the same sex was exsanguinated under isofluorane anaesthesia and serum retained for T4 analysis.

#### MMI hypo/hyperthyroid models

Twenty eight pregnant C57BL/6 mice were divided randomly into 4 groups: control, hypothyroid, hypothyroid/replacement and hyperthyroid. On PND 5 all dams were supplied with drinking water containing sucrose starting at 1% and increasing to 2% on PND 9. Dams and their litters in the hypothyroid and hypothyroid/replacement groups were rendered hypothyroid for 3 days by providing drinking water containing 0.05% MMI, 1% sodium perchlorate and 2% sucrose water starting on PND 12. On PND 15 all pups received *i.p.* injections of saline containing 2 µM NaOH (Control and hypothyroid); T4/T3 at 50 µg/5.0 µg/100 g B.W. (hyperthyroid); or T4/T3 at 25 µg/2.5 µg/100 g B.W. (hypothyroid/replacement). Pups were sacrificed by decapitation exactly 4 hrs post injection. Serum was collected from trunk blood and retained for T4 analysis. The cerebellum was rapidly dissected, frozen in liquid nitrogen and stored at −80°C.

### Chromatin immunoprecipitation (ChIP) and DNA microarrays (chip)

ChIP was performed using EZ ChIP kits (Millipore Corporation, Danvers, MA) according to the manufacturer's instructions. Briefly, cerebellum from PND 15 mouse was homogenized, with a hand-held homogenizer, in PBS containing broad-spectrum protease inhibitors and was then cross-linked with 1% formaldehyde. Nuclei were collected by adding lysis buffer after cross-linkage was stopped with glycine. To ensure that DNA fragments ranged from 300 to 600 bp, the nuclear solution was sonicated, using a Fisher 60 Sonic Dismembranator (Thermo Fisher Scientific, Nepean, ON, Canada), in an ice bath with 5× 30 sec bursts at 12% power, each separated by 30 sec periods. Six percent (about 100 µl) of the sonicated solution was store at −20°C as TI, while the remainder was incubated with anti-TRβ polyclonal antibody (PA1-213, cloneTRb-62, Affinity Bioreagents, Golden, CO) overnight with agitation at 4°C (for ChIP-PCR, half of the remainder was incubated with anti-TRβ polyclonal antibody, the other half was incubated with normal rabbit IgG (Millipore Corporation, Danvers, MA)). Antibody-bound chromatin was precipitated with protein G conjugated agarose beads, washed with gradient stringent buffers, and eluted with elution buffer as per the manufacturer's instructions. The eluted solution, as well as the stored TI, was incubated at 65°C overnight to reverse cross-links. IP DNA and TI DNA were then purified by treatment with RNase, proteinase K and multiple phenol: chloroform: isoamyl alcohol (25∶24∶1) extractions. Equivalent amounts of IP DNA and TI DNA were amplified in parallel, using a random primer method with GenomePlex Complete Whole Genome Amplification Kit (Sigma-Aldrich, Oakville, ON, Canada), according to the manufacturer's instructions (15 cycles).

Genomic regions enriched by ChIP were identified using Agilent custom microarrays (Agilent Technologies, Mississauga, ON, Canada) containing representative sequences from 5000 mouse genes selected as potential TH-regulated candidates based on our previous studies [19], [63] or from the literatures. The full list of genes is available upon request. The microarrays were prepared such that each array was composed of two slides of 44,000 spots each. Promoter oligo probes (50–60 mers) complementary to genomic sequences ranged from −8 kb upstream to 2 kb downstream of the TSS of each gene with 200 bp between adjacent probes.

Amplified IP or TI DNA samples (2 µg) was labelled with Cy5-dUTP or Cy3-dUTP (Perkin Elmer Life Sciences, Woodbridge, ON, Canada), respectively, using CGH kits (Invitrogen, Burlington, ON, Canada). Labelled DNAs (5 µg each) were hybridized with custom promoter microarrays for 40 hrs at 65°C, then washed and dried according to the manufacturer's instructions.

Hybridization images were obtained using an Agilent DNA microarray scanner and intensity data was extracted using Feature Extraction software (Agilent Technologies). Genomic regions enriched by ChIP were identified with a peak detection algorithm using Chip Analytics 1.3 software, according to the manufacturer's instructions (Agilent Technologies). Intensity data were normalized with blank subtraction followed by intra-array Lowess normalization, while the Whitehead Error Model v1.0 was used to calculate confidence values for each spot on each array. The Whitehead per-array neighbourhood model v1.0 was used to identify the bound regions. Criteria for identification of a positive probe were: 1) *P*-value for probe sets (probe and its two immediate neighbours) was less than 0.001; and 2) two of three probes in a probe set had a single probe *P*-value less than 0.005, or, the center probe in the probe set had a single probe *P*-value less than 0.001 and one of the flanking probes had a single *P*-value less than 0.1.

#### ChIP-PCR

Primers targeting the enriched regions identified with ChIP-on-chip analysis were designed using BeaconDesigner 2.0 Software (Premier Biosoft, Palo Alto, CA). PCRs were performed using AmpliTaq (Perkin Elmer) with amplified TRβ-IP, IgG-IP or TI DNA pooled of 2 independent samples as templates. Primer sequences are indicated in Table S2.

#### Expression RT-PCR

Total RNA was extracted from cerebellum using Trizol, and reverse transcribed into cDNA using SuperScript III (Invitrogen). Quantitative PCR was performed with an iCycler IQ real-time detection system (Bio-Rad Laboratories, Mississauga, ON, Canada) using SYBR-Green. Primers were designed using Beacon Designer 2.0, and sequences can be found in Table S3. Gene expression levels were normalized to *Hprt*, which was found to be stable using the DNA microarray (data not shown). PCR efficiency was examined using the standard curve for each gene. The primer specificity was determined by the melting curve for each amplicon.

#### Reporter plasmid construction

Mouse MAG luciferase reporter plasmids 1–3 were constructed by cloning PCR-derived fragments (*MAG* nucleotides +14 to −180,−280,−390) into the luciferase vector, PGL4.10 (Promega, Madisson, WI. USA). PCRs were performed using primers containing *XhoI* and *BglII* sites with mouse genomic DNA as template. PCR fragments were then subcloned into *XhoI*/*BglII* sites of pGL4.10 and the constructed sequences were confirmed by restriction enzyme mapping and sequencing.

#### Cell culture, transfection, and reporter assay

Twenty-four hrs before transfection, GH3 cells (2×10^5^) were seeded in each well of 6-well plates with F12 medium containing 10% dextran-coated charcoal-treated FBS. Each luciferase reporter plasmid construct (1.02 µg) was co-transfected with 0.03 µg of pRL-TK (Promega) into GH3 cells using 3 µl of FUGENE 6 (Agilent). Twenty four hrs post-transfection, T3 was added to a final concentration of 10^−8^ M. Cells were harvested 24 hrs after T3 addition, and then firefly and renilla luciferase activities were determined in cell lysates using a Veritas luminometer with the Dual-luciferase reporter assay system (Promega). Firefly luciferase activity was normalized to renilla luciferase activity to correct for transfection efficiency and the reporter gene expression presented as relative luciferase units (RLU). Each incubation was performed in duplicate and experiments were repeated 3 times.

#### Western blots

Lysis buffer (200 µl) was added to 1×10^6^ GH3 cells and 50 µg protein was loaded in each lane and separated on a 10% SDS-PAGE gels, then were transferred to nitrocellulose membranes at 100 V for 1 hr. Membranes were probed with anti-TRβ polyclonal antibody (Affinity Bioreagents; 3 µg/ml) overnight and HRP-conjugated goat anti-rabbit secondary antibody (Santa Cruz Biotechnology, CA; 1∶1000) for 2 hrs. Signals were detected using an ECL Plus kit (GE Healthcare Bio-Science Inc. Baie d'Urfe, QC, Canada).

#### Statistical analysis

RT-PCR gene expression and luciferase activity data are expressed as mean±S.E. Significant differences compared to control were determined using a 2-tailed Student's *t*- test and were deemed significant if *P*<0.05.

## Supporting Information

Table S1 ChIP PCR primers
(0.03 MB DOC)

Table S2 Gene expression PCR primers
(0.03 MB DOC)

Table S3 Serum T4 of mouse pups from in vivo TH modulation studies
(0.03 MB DOC)
