# Supplementary material for: Identification of Thyroid Hormone Receptor Binding Sites and Target Genes Using ChIP-on-Chip in Developing Mouse Cerebellum
Source: PLoS One. 2009 Feb 25;4(2):e4610. doi: 10.1371/journal.pone.0004610 (PMC2643481; doi:10.1371/journal.pone.0004610)
Supplement: Table S1 — ChIP PCR primers (0.03 MB DOC) [file pone.0004610.s001.doc]

Supplementary Table 1. ChIP PCR primers

| **Gene** | **Forward primer (5'-----3')** | **Reverse Primer (5'-----3')** |
| --- | --- | --- |
| CD44 | ATCTGCTGATGTGGATGTG | CTTCCGTTGGCTGCTTAG |
| CD81 | TTCCCTGGAGATTCTACC | CTCTGGCTTCTCTAATGG |
| FIGN | ATGACTTTATCTGCTAGG | TCTTAAACCTTATGTTGC |
| GTF3c1 | GTTCTGCATCGCACCATC | CGGGCATCAGTTTCTACG |
| HES5 | GCTGCTCTCTGACACCAC | ATATCCCATCTCCAAAACTTCC |
| IGF-2 | CAGTTAGAGGGTTACAAGG | CTATCTGGCTGCTATTCG |
| LMO2 | TGGATGATTCGCTCTCTC | TTACTCAGACGGGTCCAG |
| MAG | TTCCTGGGTCCTACTAGC | CATCTCTGAGTGCCAAGG |
| Golli-MBP | GTAGACACCAGTTTCGAT | CTGTGGGCACTGCCTGGC |
| PAX3 | TCTGTATATGTTGGTGAGG | TACTCATCTCAACGATAGG |
| RXR | CCTGGGTAGGAGTGCTTC | ACACATGGGGAAACTGAGG |
| SMS | CAAGAACCTAACACTGACG | CCTTTGCAAGGTGAAGTAC |
| VLDLr | TGCTTTTCTTTCTCCCATCC | ACACACAGGGCTCCAAAG |
|  |  |  |
