# Supplementary material for: Identification of Thyroid Hormone Receptor Binding Sites and Target Genes Using ChIP-on-Chip in Developing Mouse Cerebellum
Source: PLoS One. 2009 Feb 25;4(2):e4610. doi: 10.1371/journal.pone.0004610 (PMC2643481; doi:10.1371/journal.pone.0004610)
Supplement: Table S2 — Gene expression PCR primers (0.03 MB DOC) [file pone.0004610.s002.doc]

Supplementary Table 2. Gene expression PCR primers

| Gene | Forward primer (5'-----3') | Reverse Primer (5'-----3') |
| --- | --- | --- |
| GTF3c1 | GTGGTGTCCCTTCCCTTGC | TCATCATCGTGGTCGTCTTCC |
| MAG | GCTACACTTCGTGCCTAC | AATTCATCTCCACAATCACTG |
| LMO2 | GAGTGGACCAAGATCAATG | CCATAATCTCCTAAGAAATGC |
| SMS | GAGTGACTTGGCATATACC | CAATCTCTACCATAGTGACC |
