# Supplementary material for: Identification of Thyroid Hormone Receptor Binding Sites and Target Genes Using ChIP-on-Chip in Developing Mouse Cerebellum
Source: PLoS One. 2009 Feb 25;4(2):e4610. doi: 10.1371/journal.pone.0004610 (PMC2643481; doi:10.1371/journal.pone.0004610)
Supplement: Table S3 — Serum T4 of mouse pups from in vivo TH modulation studies (0.03 MB DOC) [file pone.0004610.s003.doc]

Supplementary Table 3. Serum T4 of mouse pups from *in vivo* TH modulation studies

| Treatment (Exp 1) | n | Serum Thyroxine (ng/dl) |
| --- | --- | --- |
| Vehicle | 5 | 9.9 ± 1.27 ^b^ |
| + high T4/T3 | 5 | 51.1 ± 27.2 ^d^ |
| MMI + Perc | 5 | 1.9 ± 0.5 ^a^ |
| MMI + Perc+ Low T4/T3 | 4 | 16.6 ± 5.6 ^c^ |
|  |  |  |
| Treatment (Exp 2) |  |  |
| Vehicle | 5 | 9.67 ± 0.55 ^b^ |
| PTU | 5 | 0.44 ± 0.17 ^a^ |

^abcd^ Means displaying the same letter superscript are not significantly different (p> 0.05)

Hypothyroid and hyperthyroid mouse models were established as described in Material and Methods. Pups were sacrificed by decapitation and trunk blood serum collected and stored for subsequent T4 analysis (thyroxine RIA kit, MP Biomedicals, Solon, OH).

Statistical analyses: Serum T4 levels were log transformed prior to statistical analyses as normality (Kolmogorov-Smirnov test) and homogeneity of variance tests (Levene Median test) failed for data from both experiments. Data from the PTU study were analysed by one tailed t-test while levels from the MMI/Perc study were analysed by ANOVA followed by Holm-Sidak post hoc analysis. All tests were performed using the Statistical modules of SigmaPlot v.11 (Systat Software, Chicago, IL).
